# Effect of Resistance Exercise on the Lipolysis Pathway in Obese Pre- and Postmenopausal Women

**DOI:** 10.3390/jpm11090874

**Published:** 2021-08-31

**Authors:** Sunghwun Kang, Kyu-Min Park, Kun-Young Sung, Yuning Yuan, Seung-Taek Lim

**Affiliations:** 1 Laboratory of Exercise Physiology, Department of Sport Science, College of Art, Culture and Engineering, Kangwon National University, Chuncheon-si 24341, Gangwon-do, Korea; 94psycho@kangwon.ac.kr (S.K.); yuanyuning80@gmail.com (Y.Y.); 2 Interdisciplinary Program in Biohealth-Machinery Convergence Engineering, Kangwon National University, Chuncheon-si 24341, Gangwon-do, Korea; 3 Center for Sports Science in Gangwon, Chuncheon-si 24239, Gangwon-do, Korea; katc-kyumin@kangwon.ac.kr; 4 Department of Plastic and Reconstructive Surgery, College of Medicine, Kangwon National University Hospital, Kangwon National University, Chuncheon-si 24289, Gangwon-do, Korea; ps@kangwon.ac.kr; 5 Olympic Studies Center, Kangwon National University, Chuncheon-si 24341, Gangwon-do, Korea

**Keywords:** exercise, lipolysis, menopause, obesity, women, resistance training

## Abstract

Physical exercise may stimulate lipolytic activity within adipose tissue. Furthermore, resistance exercise may contribute to the more efficient reduction in adipose tissue mass and prevent the accumulation thereof in obese women. The purpose of this study was to examine the effects of regular resistance exercise for 12 weeks on the lipolysis pathway in women with obesity. Twenty-three pre- and postmenopausal women with body fat percentages of 30% or more were divided into the premenopausal group (*n* = 9) and the postmenopausal group (*n* = 14). All subjects participated in resistance exercise training for 12 weeks. Anthropometric and physical fitness tests were performed on all participants. Protein analyses were performed on extracted subcutaneous fatty tissue, and changes in the relevant protein levels in the samples were analyzed by Western blotting. All serum samples were submitted for enzyme-linked immunosorbent assay measurements of adipocyte factors. After 12 weeks, the adipose triglyceride lipase, monoacylglycerol lipase, and perilipin1 protein levels were significantly lower in the postmenopausal group than in the premenopausal group. The hormone-sensitive lipase protein levels were significantly higher in the postmenopausal group than in the premenopausal group. In addition, leptin concentrations were significantly decreased after resistance exercise in the postmenopausal group. Adiponectin concentrations were significantly increased after resistance exercise in both groups. These findings indicate that regular resistance exercise is effective in reducing the weight and body fat of obese premenopausal women, and in the secretion of adiponectin. On the other hand, postmenopausal women were found to have redeced weight and body fat, and were found to be positive for the secretion of adipokine factors. In addition, positive changes in lipolysis pathway factors in adipose tissue promote lipid degradation and reduce fat mass. Thus, regular resistance exercise shows positive changes in the lipolysis pathway more effectively in weight and body fat reduction in postmenopausal women than in premenopausal women.

## 1. Introduction

Adipose tissue is recognized as an endocrine organ. A complex interplay between multiple endocrine mediators and the sympathetic nervous system has been shown to govern adipocyte metabolism. In the postprandial state, insulin promotes glucose and fatty acid uptake, as well as lipogenesis, and suppresses triglyceride lipolysis [1].

Excess body weight results from an imbalance between energy intake and energy expenditure [2]. One way to maintain the correct body weight is to stimulate lipid catabolism through increased physical activity. Properly designed training simulates fat breakdown, that is, the hydrolysis of triacylglycerol stored in adipose tissue, releasing free fatty acids into the circulation and causing oxidation in muscles and other tissues [3]. Some studies have demonstrated that low-intensity endurance training leads to maximal lipid oxidation. However, the available evidence for this is inconclusive [4]. The post-exercise decrease in triacylglycerols content in adipose tissue is a consequence of enhanced lipolysis. The process, initiated by adipose triglyceride lipase (ATGL), is then continued by hormone-sensitive lipase (HSL), and eventually, the last free fat chain is hydrolyzed by monoacylglycerol lipase (MGL) [5]. Moreover, perilipin1 (PLIN1) is highly expressed in adipose tissue and plays an important role in controlling basic lipolysis and stimulating lipolysis [6]. PLIN1 co-localizes and interacts in cultured human primary adipocytes and regulates the lipid droplet size in human adipocytes in a cooperative manner [7]. 

Chronic exercise was shown to normalize the markers of this process, phosphorylated HSL and ATGL, in obese humans [8]. In addition, the lipolysis response was reduced in obese individuals, which can be attributed to a decrease in the intracellular activation of fat breakdown in fat cells to some extent [9]. A recent study demonstrated that triacylglycerol lipase activity increased 16-fold at 10 min of acute resistance exercise in obese men [10]. Furthermore, the different types of muscle fibers influenced expressed high ATGL and HSL in healthy humans [11]. 

Adipose tissue is a major endocrine organization that produces and secretes a wide range of intermediates to regulate adipocytes differentiation and de-differentiation [12]. Leptin and adiponectin are hormones released by adipocytes in adipose tissue. Increased circulating leptin levels may also contribute to enhancing basal lipolysis in obesity [13]. The reduction in lipolysis associated with low adiponectin expression in obese patients may contribute to the progression of obesity by reducing the precise recruitment of adipose tissue fatty acids to muscles or the liver [14].

Changes in the metabolism of adipose tissue can greatly contribute to changes in body fat distribution during the menopause transition, and the menopausal condition might also be affected by adipose tissue lipolysis, which, in turn, can contribute to changes in body composition [15]. A previous study observed greater lipolytic reactions and sensitivity in the abdominal, breast, and femoral adipose tissue of postmenopausal women compared to premenopausal women, but the effects were not seen in the cells of postmenopausal females [16]. However, resistance exercises have been effective in improving body composition in pre and postmenopausal women [17]. Acute resistance exercise increased subcutaneous abdominal adipose tissue lipid decomposition and fatty oxidation in women [18].

Thus, physical exercise may stimulate lipolytic activity within adipose tissue. Furthermore, resistance exercise may contribute to a more efficient reduction in adipose tissue mass and prevent its accumulation. However, there is a lack of research that directly analyzes adipose tissue in humans and long-term resistance exercise intervention.

The purpose of this study was to investigate the modulation of 12 weeks of regular resistance exercise on the lipolysis pathway in obese pre and postmenopausal women. In this study, it was hypothesized that obese pre and postmenopausal women will have different lipolysis pathways due to regular resistance exercise, and there will be differences in the concentrations of leptin and adiponectin. 

## 2. Methods

### 2.1. Participations

The forty participants were arbitrarily enrolled from the general population of local communities. To be eligible for inclusion, the participants had to meet the following inclusion criteria: (1) postmenopausal (absence of a menstrual cycle for at least one year and follicle-stimulating hormone level of >30 IU/L) and at least 40 (premenopausal) and 50 (postmenopausal) years of age on the date of the assessment, (2) a body fat percentage of 30% or more on the date of the assessment, (3) not receiving hormone replacement treatment, and (4) not using drugs such as beta-blockers and statins. If the participation inclusion criteria were not met, they were excluded. The final sample of 23 women was divided into the premenopausal group (PRE, *n* = 9) and the postmenopausal group (POST, *n* = 14). The participant sample size was calculated using ANOVA with a large effect size of 0.90, a significance level of 0.05, and a power of 0.80 (G*power 3.2.1). 

All volunteers underwent medical screening by a medical specialist, including a health status interview and physical examination. Written informed consent was obtained from all subjects. The study was approved by the Kangwon National University Institutional Review Board (KWNUIRB-2016-04-009-002) and conducted in agreement with the Declaration of Helsinki.

The characteristics of the participants are shown in Table 1.

### 2.2. Body Composition

Anthropometric measurements were collected on all participants by using the protocol described by Park et al. [19]. Body composition was measured using a multi-frequency bioelectrical impedance analyzer with eight tactile electrodes (MF- BIA8) (Inbody 720 Body Composition Analyzer, Biospace, Seoul, Korea) at the Exercise Physiology Laboratory of Kangwon National University. Bioelectrical impedance analysis was performed after at least 8 h of fasting and voiding. This analyzer uses an alternating current of 250 mA at multiple frequencies of 1 kHz, 5 kHz, 50 kHz, 250 kHz, 500 kHz and 1000 kHz. It measures segmental impedances at the right arm, left arm, right, leg, left leg and trunk for all frequencies. The body mass index (BMI) was calculated as weight in kilograms divided by the square of height in meters. The waist-to-hip ratio (WHR) was calculated as waist circumference divided by the hip circumference. Arterial blood pressure was measured by a mercury sphygmomanometer after the participants had been seated at rest for 10 min. Two measurements were taken at each time point, and the mean was calculated and used for analysis.

### 2.3. Subcutaneous Fatty Tissue Extraction and Western Blots

All study participants agreed to an abdominal biopsy.

The plastic surgeon extracted 30 g of abdominal fat twice, once before and once after the exercise program, as previously described by Hirsch et al. [20]. On the first time, right-side abdominal fat was extracted. On the second time, left-side abdominal fat was extracted after the exercise program. The participants lied on the operating bed and a plastic surgeon cleaned the participant’s abdomen with betadine. The plastic surgeon anesthetized the incisional window (1 cm length) for the liposuction machine tip with 2% lidocaine. The incisional window was made by a number 15 blade, and tumescent solution (500 cc of saline, 2% lidocaine 5 cc and 0.1 cc of epinephrine were mixed) was infiltrated around the incisional window. The plastic surgeon extracted abdominal fat (30 cc) using a liposuction machine according to a previous report by Hirsch and Gallian [21]. The fat was centrifuged for three minutes to separate the pure fat cells. The pure abdominal fat was stored at –18 °C immediately. The incisional window was sutured with 4-0 nylon and covered with a waterproof bandage. The suture materials were removed at seven postoperative days.

To extract protein from the subcutaneous fatty tissue, the tissues were lysed in 200 μL of radioimmunoprecipitation assay (RIPA) buffer. The tissue was homogenized and centrifuged for 30 min at 14,000 RPM. The protein concentration of the supernatant was measured using the BCA Protein Assay Kit (Pierce, Rockford, IL, USA). Samples of equal protein content were resolved by sodium dodecyl sulfate (SDS) polyacrylamide gel electrophoresis on 10% gels and transferred to a polyvinylidene fluoride (PVDF) membrane. The membrane was blocked with 5% skim milk in 1x Tris-buffered saline with 0.1% Tween^®^ 20 detergent (TBST), and subsequently incubated at 4 °C overnight with primary antibodies (1:1000 dilution) against perilipin1 (PLIN1) (sc-47320), adipose triglyceride lipase (ATGL, sc-50223), monoglyceride lipase (MGL, sc-398942), and hormone-sensitive lipase (HSL, sc-25843) (all from Santa Cruz Biotechnology Inc., Santa Cruz, CA, USA). Subsequently, the membranes were incubated with an appropriate secondary antibody (1:5000 dilution) conjugated with donkey anti-goat IgG (PLIN1 and ATGL), goat anti-rabbit IgG (HSL), and goat anti-mouse IgG (MGL) for 1 h at room temperature. β-actin (sc-28365, goat anti-mouse IgG) expression was confirmed through an experimental process from blocking after each factor was checked and stripped. The signal was developed with an enhanced chemiluminescence solution (Amersham Pharmacia Biotech Inc., Piscataway, NJ, USA) and visualized using the Image Quant TM LAS-4000 System (GE Healthcare, Uppsala, Sweden). Relative protein band intensities were quantified using image J software version 1.52a (National Institute of Health, MD, USA), and the values were normalized to the β-actin band. Arbitrary units were normalized on the expression of the housekeeping protein beta-actin.

### 2.4. Blood Collection and Analysis

Fasting venous blood samples were collected from all participants at baseline, 6 weeks, and 12 weeks by using the protocol described by Park et al. [19]. Fasting was maintained for at least 8 h, and blood samples were collected on the following day. The participants were advised to get enough sleep and refrain from radical movements as much as possible. All samples were taken from an antecubital vein at 0830 AM. Serum samples were obtained after centrifugation and stored at −80 °C. Serum levels of leptin and adiponectin were measured using enzyme-linked immunosorbent assay DuoSet Kits (R&D Systems, Minneapolis, MN, USA) according to the manufacturer’s instructions, as described previously.

### 2.5. Exercise Intervention

The resistance exercise program was applied as in the experiments performed by Gurudut and Rajan [22], with modifications to fit the purpose of our experiment. The goal of the resistance exercise intervention was to perform moderate-intensity exercise 60 min per day, three days per week for 12 weeks under supervision. At the beginning of each session, there was 10 min of warm-up. This was followed by 40 min of main exercises with specific content and 10 min of cool-down. The warm-up exercises included five minutes of stretching, and five minutes of power walking at 50% intensity of the maximal heart rate reserve. Among the resistance exercises, moderate-intensity exercise was defined as circuit exercises at 55–65% intensity of one repetition maximum (1-RM) with a 230–260 kcal burn, 12 repetitions, and three sets. There was a rest period between each category of 30 s. The rest period between sets was one minute in the total resistance exercise time of 60 min. The participants performed the same five lower and upper body resistance exercises (upper-body resistance exercises: chest press (KW-021, KAIROS, Gimpo, Korea), let pull-down (KW-002, KAIROS, Gimpo, Korea), biceps curl (KW-006, KAIROS, Gimpo, Korea), triceps extension (KW-016, KAIROS, Gimpo, Korea), and crunch; lower-body resistance exercises: squat (KW-012, KAIROS, Gimpo, Korea), lunge, knee extension (KW-009, KAIROS, Gimpo, Korea), and calf raise (KW-012, KAIROS, Gimpo, Korea)). All exercise groups were given a polar (heart rate monitor; M400, Kempele, Finland) portable exercise intensity setting device for 60 min. The measurement of 1 RM was calculated using the formula of Brzycki: 1 RM = lifted weight (lb)/(1.0278 − repetitions × 0.0278). All exercises were performed by re-measuring 1 RM every two weeks [23].

Dietitians and exercise physiologists also met regularly with a clinical health psychologist experienced in lifestyle behavior change to discuss the participant’s progress and refine behavior modification goals (pain during exercise, side-effects due to weight loss, and encouragement to participate in exercising) according to each participant’s needs. Nutritional education, self-management exercise, and behavior change techniques were provided. Telephone consultations were scheduled biweekly for monitoring and motivation.

### 2.6. Statistical Analysis

The data were analyzed using the SPSS 22.0 for Windows computer software package and expressed as the mean and standard deviation. All data were tested for normal distribution using the Shapiro-Wilk test. The independent samples t-test was used to assess group differences in the baseline variables. A two-way analysis of variance (ANOVA) was used to determine the interactions of group (PRE vs. POST) and time (baseline, 6 weeks, and 12 weeks) effects on body composition, physical fitness, and adipokine variables. Bonferroni’s correction with a paired t-test test was used for post hoc analysis. Adipose tissue levels between the groups before and after 12 weeks were analyzed by t-test (independent and paired). Statistical significance was accepted at the 0.05 level.

## 3. Results

### 3.1. Changes in Body Composition after Exercise

The changes in body composition in each group are shown in Table 2. The two-way factor ANOVA results showed no significant group × time interaction. Weight (main effect of time, *p* < 0.01), BMI (main effect of time, *p* < 0.001), percent fat (main effect of time, *p* < 0.001), WHR (main effect of time, *p* < 0.001), and SBP (main effect of time, *p* < 0.01) were significantly decreased from the baseline values after 12 weeks.

Post hoc analysis using Bonferroni’s correction with a paired t-test indicated significant decreases in the PRE group in weight, percent fat, BMI, WHR, SBP, and DBP at 12 weeks compared to 6 weeks and baseline. Weight, BMI, percent fat, and WHR were decreased at 12 weeks compared to 6 weeks and baseline in the POST group. 

### 3.2. Changes in Adipokines after Exercise

The changes in adipokines in each group are shown in Table 3. The two-way factor ANOVA results showed no significant group × time interaction. Leptin (main effect of time, *p* < 0.01) was significantly decreased after 12 weeks compared to the baseline values. Adiponectin (main effect of time, *p* < 0.001) was significantly increased after 12 weeks compared to the baseline values.

Post hoc analysis using Bonferroni’s correction with a paired t-test in the POST group indicated significant decreases in leptin concentrations at 12 weeks compared to the 6-week and baseline values. The adiponectin concentrations were greater in both groups at 12 weeks compared to the 6-week and baseline values.

### 3.3. ATGL, HSL, MGL, and PLIN1 Levels in Subcutaneous Fatty Tissue

**Figure 1:**
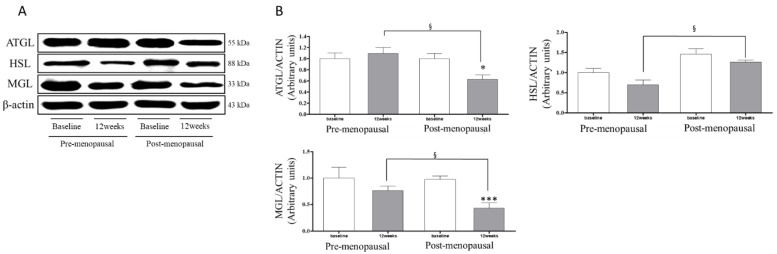
Changes in ATGL, HSL, and MGL levels in pre and postmenopausal women. (**A**) Western blot analysis of ATGL, HSL, and MGL in adipose tissue from participant’s abdominal fat. (**B**) Changes in ATGL, HSL, and MGL levels in subcutaneous fatty tissue for the premenopausal and postmenopausal groups at baseline and post-12 weeks. Data in the bar graph represent the means ± standard deviation. * *p* < 0.05, *** *p* < 0.001; significantly different from baseline in the within-group. *p*-value was analyzed by paired *t*-test. § *p* < 0.05; significantly different from 12 weeks in the between group. *p*-value was analyzed by independent *t*-test.

Figure 1 shows the ATGL, HSL, and MGL levels changed in the subcutaneous fatty tissue in the two groups at baseline and post-12 weeks. The within-group analysis showed that the ATGL protein levels were significantly decreased in the POST group after 12 weeks relative to the baseline values (−37.15%, *p* < 0.05), but no significant change was observed in the PRE group. After 12 weeks, the ATGL protein level was 42.51% lower in the POST group than in the PRE group.

The HSL levels changed in the subcutaneous fatty tissue in the two groups at baseline and post-12 weeks (Figure 1). After 12 weeks the HSL protein level was 79.27% significantly higher in the POST group than in the PRE group. However, there was no significant difference in the within-group comparison.

The MGL levels changed in subcutaneous fatty tissue in the two groups at baseline and post-12 weeks. The within-group analysis showed that the MGL protein levels were significantly decreased in the POST group after 12 weeks relative to the baseline values (−56.34%, *p* < 0.001), but no significant changes were observed in the PRE group. After 12 weeks the MGL protein levels were 43.21% significantly lower in the POST group than in the PRE group.

**Figure 2:**
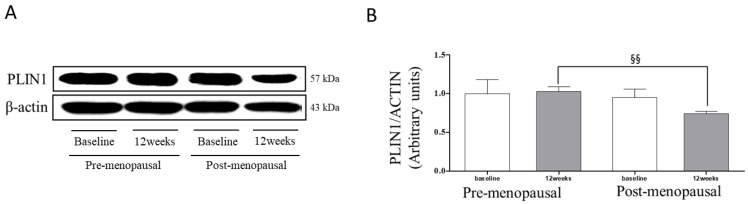
Changes in PLIN1 levels in pre and postmenopausal women. (**A**) Western blot analysis of PLIN1 in adipose tissue from participant’s abdominal fat. (**B**) Changes of PLIN1 levels in subcutaneous fatty tissue for the premenopausal and postmenopausal groups at baseline and post 12 weeks. Data in the bar graph represent the means ± standard deviation. §§ *p* < 0.01; significantly different from 12 weeks in the between group. *p*-value was analyzed by independent *t*-test.

Figure 2 shows the PLIN1 levels change in the subcutaneous fatty tissue in the two groups at baseline and post-12 weeks. After 12 weeks, the PLIN1 protein levels were 27.50% significantly lower in the POST group than in the PRE group. However, there was no significant difference in the within-group comparison.

## 4. Discussion

This study investigated the modulation of regular resistance exercise on the lipolysis pathway in pre- and postmenopausal women. The main finding of this study was that after 12 weeks, ATGL, MGL, and PLIN1 protein levels were lower by 42.51%, 43.21%, and 27.50%, respectively, in the POST group compared to the PRE group, and the HSL protein levels were higher by 79.27% in the POST group than in the PRE group. In addition, leptin concentrations were significantly decreased after resistance exercise in both groups, and adiponectin concentrations were significantly increased after resistance exercise in both groups.

Post- menopausal women had higher amounts of intramuscular fat and subcutaneous adipose tissue compared to men and premenopausal women, and the activity of key enzymes involved in free fatty acid (FFA) metabolism was different between pre and postmenopausal women [24,25]. The relationship of FFA release, originating from adipose tissue triglyceride fatty acid, to the amount of body fat represents the only route by which these fat stores can be transported through oxidation to nonadipose tissue for a net loss [26]. Triacylglycerol and diacylglycerol are degraded by the lipases ATGL and HSL, and fasting lipolysis, expressed per unit of fat mass, may be reduced in obese patients [27]. Diacylglycerol is converted to monoacylglycerol and a second fatty acid by the action of HSL, after which MGL hydrolyzes monoacylglycerol to produce glycerol and the last fatty acid [28]. PLIN1 protein is thought to modulate the access of HSL to the surface of fat droplets [29], which plays a major role in lipolysis by regulating the function of lipases [30]. In this study, changes in lipolysis-related lipases such as ATGL, HSL, MGL, and PLIN1 were observed after 12 weeks of resistance exercises in pre and postmenopausal obese women. The ATGL and MGL levels were significantly decreased by 37.15% and 56.34%, respectively, after 12 weeks of resistance exercise in the POST group. Cyclic adenosine monophosphate (cAMP) produced by adenylate cyclase activates protein kinase A (PKA) and PKA phosphorylates and activates two or more substrates, including HSL and PLIN1 [31]. HSL and PLIN1 phosphorylation leads to the translocation of HSL from the cytosol to the surface of lipid droplets, and insulin can activate protein phosphatases, resulting in the subsequent dephosphorylation of HSL in this case, and ATGL instead of phosphate and translation [31]. Estrogen and estrogen receptor alpha are known to repress intra-abdominal adipose formation [32]. Wend et al. [33] measured the lipid droplet size (relative area per lipid droplet) and the number of lipid droplets per cell in estrogen receptor alpha knockout (ERαKO) postmenopausal model mice, and reported that ATGL levels and lipid droplets in the ERαKO cells were significantly decreased compared to wild-type model mice. These results indicated that ERαKO mice developed more adipose tissue in the perirenal, periovarian, and mesenteric/omental regions than wild-type model mice did [34]. Walhin et al. [35] reported that HSL was decreased in the positive energy balance period and HSL was increased in the negative energy balance period. However, other studies have shown that lipolysis activation occurred through increased levels of ATGL, HSL, and MGL and glycerol release [36]. In a pervious study, the ATGL, HSL, and MGL activities were significantly higher in the diet and exercise mice group than in the high-fat diet-fed mice group [37]. In this study, the ATGL and MGL levels were significantly decreased after 12 weeks of resistance exercise. The HSL and PLIN1 levels tended to decrease more after 12 weeks of resistance exercise in the POST group than the PRE group, which is similar to Wend et al. [33] and Walhin et al. [35]. After 12 weeks of resistance exercise, the total adipose tissue may be reduced due to a significant decrease in body weight and body fat percentage. In addition, a greater decrease in lipolysis factors in the POST group seemed to have a greater effect on resistance exercise than in the PRE group. 

Moreover, leptin concentrations were significantly reduced at 12 weeks compared to 6 weeks and baseline, and adiponectin concentrations were higher at 12 weeks compared to 6 weeks and baseline in the POST group compared to the PRE group, supporting lipolysis by ATGL, HSL, MGL, and PLIN1.

Adipocytes in adipose tissue release several polypeptides, as well as free fatty acids, which are products of lipolysis, also known as adipocytokines, leptin, and adiponectin [38]. In humans, plasma adiponectin concentrations decrease with obesity, and plasma leptin concentrations are highly correlated with BMI [39]. Adiponectin is an adipocyte-derived hormone that sensitizes insulin and improves the energy metabolism of tissues [40]. In this regard, adiponectin is relatively unique as an adipokine because it is expressed at the highest levels in lean and healthy individuals [41]. Several studies have reported the impact of resistance exercise on women. Park et al. [19] showed significant differences in leptin and adiponectin levels in pre- and postmenopausal women after 12 weeks of resistance exercise, indicating similar results to this study. Dieli-Conwright et al. [42] showed that leptin, adiponectin, and BMI were significantly improved after 16 weeks of aerobic and resistance exercise in overweight or obese survivors of breast cancer. Rosety-Rodriguez et al. [43] reported that leptin levels were significantly decreased after the completion of the resistance circuit training. In the above studies, regular resistance exercises improved leptin and adiponectin levels and reduced the body fat percentage in both groups. The concentration control idealization of adipokines (leptin and adiponectin), lipolysis lipases (ATGL, HSL, and MGL), and PLIN1 in adipose tissues that cause inflammation by cholesterol contributed to increases in fat decomposition and increases in cyclic nonesterified fatty acids [44]. In this study, regular resistance exercise reduced leptin levels, body weight, and fat percentages, and increased adiponectin levels. The reason for the reduction in total adipose tissue is supported by decreased ATGL, HSL, MGL, and PLIN1 levels in subcutaneous fatty tissue after 12 weeks of resistance exercise

Resistance exercise is well-known to improve body composition in obese adults [45]. Resistance exercise was shown to increase fat free mass and decrease body weight in pre and postmenopausal women with obesity [46,47]. This study found that body weight, BMI, fat percentage, and the waist-to-hip ratio were significantly decreased after 12 weeks of resistance exercise compared to 6 weeks in both groups. Weight gain and obesity largely drive the increased prevalence of metabolic syndrome in pre- and postmenopausal women [48]. Because of the associated sequelae of coronary heart disease, cardiovascular disease, diabetes, and mortality, lifestyle management should be of paramount importance in obese women.

Finally, this study meaningfully conducted direct analyses of adipose tissue in humans. However, the present study had some limitations. The sample size was small, which limits our ability to determine the significance of the results. Therefore, additional studies with larger sample sizes and a control group are required to determine the effectiveness of regular resistance exercise on lipolysis. Another limitation was that gender and age differences in the lipolysis factors were not analyzed. In addition, we did not control for confounding factors such as food energy intake and habitual activities. Lipolysis reactions and lipid storage for food energy intake and habitual activity are key factors in determining body fat stores. Therefore, confounding factors should be considered in future studies.

## 5. Conclusions

In conclusion, the results of this study indicated that regular resistance exercise is effective in reducing the weight and body fat of obese premenopausal women, and in the secretion of adiponectin. On the other hand, post-menopausal women were found to have reduced weight and body fat, and were found to be positive for the secretion of adipokine factors. In addition, positive changes in lipolysis pathway factors in adipose tissue promote lipid degradation and reduce fat mass. Thus, regular resistance exercise shows positive changes in the lipolysis pathway more effectively in weight and body fat reduction in postmenopausal women than in premenopausal women.

## Figures and Tables

**Table 1:** Characteristics of the participations.

Variable	Group	Mean ± SD (Range)	*p*-Value
Age (year)	PRE (*n* = 9)	44.44 ± 3.50 (41–47)	<0.001
	POST (*n* = 14)	60.50 ± 6.12 (56–64)	
Height (cm)	PRE (*n* = 9)	158.71 ± 5.28 (154–167)	0.190
	POST (*n* = 14)	155.95 ± 3.48 (151–164)	
Weight (kg)	PRE (*n* = 9)	66.90 ± 13.40 (48–83)	0.313
	POST (*n* = 14)	61.81 ± 6.51 (50–72)	
Fat (%)	PRE (*n* = 9)	36.98 ± 7.41 (31–42)	0.512
	POST (*n* = 14)	35.04 ± 5.59 (31–38)	

Mean ± SD. *p*-value was analyzed by independent *t*-test.

**Table 2:** Changes in body composition.

Variable		0 Weeks	6 Weeks	12 Weeks	F-Value (*p*-Value)
Weight (kg)	PRE (*n* = 9)	66.90 ± 13.40	66.47 ± 12.83	64.46 ± 11.73 ^a,c^	G: 1.513 (0.232) T: 10.692 (0.001) G×T: 2.463 (0.097)
	POST (*n* = 14)	61.81 ± 6.51	60.98 ± 6.14 ^b^	60.65 ± 5.68 ^a^	
BMI (kg/m^2^)	PRE (*n* = 9)	26.36 ± 4.29	26.33 ± 4.40	25.46 ± 3.74 ^c^	G: 0.392 (0.538) T: 10.606 (<0.001) G×T: 2.624 (0.084)
	POST (*n* = 14)	25.49 ± 2.69	25.10 ± 2.56 ^b^	24.96 ± 2.44 ^a^	
Muscle (kg)	PRE (*n* = 9)	22.72 ± 4.10	23.16 ± 4.03	23.26 ± 4.22	G: 0.884 (0.358) T: 2.144 (0.130) G×T: 0.438 (0.619)
	POST (*n* = 14)	21.68 ± 1.95	22.10 ± 2.06	21.86 ± 1.87	
Fat (%)	PRE (*n* = 9)	36.98 ± 7.41	35.48 ± 7.08 ^b^	33.57 ± 6.49 ^a^	G: 0.407 (0.530) T: 10.719 (<0.001) G×T: 1.958 (0.155)
	POST (*n* = 14)	35.04 ± 5.59	32.87 ± 6.03 ^b^	33.20 ± 5.37 ^a^	
WHR	PRE (*n* = 9)	0.93 ± 0.06	0.92 ± 0.06 ^b^	0.90 ± 0.05 ^a,c^	G: 3.133 (0.091) T: 14.601 (<0.001) G×T: 1.855 (0.168)
	POST (*n* = 14)	0.90 ± 0.04	0.87 ± 0.04 ^b^	0.88 ± 0.03 ^a^	
SBP (mmHg)	PRE (*n* = 9)	132.44 ± 17.56	126.44 ± 13.28	115.78 ± 12.16 ^a,c^	G: 3.565 (0.073) T: 8.754 (0.001) G×T: 1.270 (0.291)
	POST (*n* = 14)	139.00 ± 15.63	135.79 ± 14.85	131.43 ± 16.49	
DBP (mmHg)	PRE (*n* = 9)	81.00 ± 6.86	82.67 ± 7.45	76.22 ± 7.64 ^a,c^	G: 2.585 (0.123) T: 3.609 (0.071) G×T: 1.667 (0.206)
	POST (*n* = 14)	83.07 ± 7.74	87.93 ± 9.40	83.50 ± 8.34	

Mean ± SD, BMI; body mass index, WHR; waist-to-hip ratio, SBP; systolic blood pressure, DBP; diastolic blood pressure, G; Group, T; Time, G×T; Group×Time; post-hoc: ^a^: 0 week vs. 12 weeks; ^b^: 0 week vs. 6 weeks; ^c^: 6 weeks vs. 12 weeks.

**Table 3:** Changes in adipokines.

Variable		0 Weeks	6 Weeks	12 Weeks	F-Value (*p*-Value)
Leptin (pg/mL)	PRE (*n* = 9)	386.2 ± 103.9	347.7 ± 116.1	293.6 ± 155.1	G: 0.519 (0.479) T: 7.022 (0.002) G×T: 0.247 (0.782)
	POST (*n* = 14)	368.1 ± 73.9	304.0 ± 87.7 ^b^	281.7 ± 91.7 ^a^	
Adiponectin (pg/mL)	PRE (*n* = 9)	59.04 ± 4.78	62.99 ± 5.88 ^b^	66.38 ± 5.51 ^a,c^	G: 0.117 (0.736) T: 53.389 (<0.001) G×T: 1.710 (0.193)
	POST (*n* = 14)	58.98 ± 3.06	62.60 ± 4.75 ^b^	68.80 ± 6.04 ^a,c^	

Mean ± SD, G; Group, T; Time, G×T; Group×Time; post-hoc: ^a^: 0 week vs. 12 weeks; ^b^: 0 week vs. 6 weeks; ^c^: 6 weeks vs. 12 weeks.

## Data Availability

The datasets analysed during the current study are available from the corresponding author on reasonable request.
